# Neutrophil-lymphocyte ratio change after curative gastrectomy for gastric cancer: a subgroup analysis

**DOI:** 10.31744/einstein_journal/2020AO4860

**Published:** 2019-11-14

**Authors:** Daniel José Szor, André Roncon Dias, Marina Alessandra Pereira, Marcus Fernando Kodama Pertille Ramos, Bruno Zilberstein, Ivan Cecconello, Ulysses Ribeiro

**Affiliations:** 1 Universidade de São Paulo Faculdade de Medicina Instituto do Câncer do Estado de São Paulo São PauloSP Brazil Instituto do Câncer do Estado de São Paulo, Faculdade de Medicina, Universidade de São Paulo, São Paulo, SP, Brazil.

**Keywords:** Stomach neoplasms, Prognosis, Biomarkers, Inflammation, Gastrectomy

## Abstract

**Objective:**

To evaluate the impact of neutrophil-lymphocyte ratio change after curative surgery for gastric cancer.

**Methods:**

A retrospective analysis of patients with gastric cancer who underwent curative surgery between 2009 and 2017 was performed. A cutoff value was established for the neutrophil-lymphocyte ratio in the pre- and postoperative periods, according to the median value, and four subgroups were formed (low-low/low-high/high-low/high-high). Clinical-pathological and survival data were analyzed and related to these subgroups.

**Results:**

A total of 325 patients were included in the study. The cutoff values of the neutrophil-lymphocyte ratio were 2.14 and 1.8 for the pre and postoperative periods, respectively. In patients with stages I and II, the high-high subgroup presented worse overall survival (p=0.016) and disease-free survival (p=0.001). Complications were higher in the low-high subgroup of patients.

**Conclusion:**

The neutrophil-lymphocyte ratio is a low cost, efficient and reproducible marker. The prognosis individualization can be performed according to the identification of subgroups at a higher risk of complications and worse prognosis.

## INTRODUCTION

Gastric cancer (GC) is the fifth most common malignancy and the third leading cause of cancer death worldwide.^(^^1^^)^ It is known that patients with the same disease stage may present different outcomes, suggesting that several factors may contribute to the prognosis. The relation between inflammation and cancer is described since the 19^th^ century,^(^^2^^)^ and the utilization of inflammatory biomarkers to refine prognosis was proposed in the last decade.^(^^3^^,^^4^^)^ Tumoral microenvironment is formed by multiple types of cells, including neutrophils and lymphocytes, which play specific roles in the inflammatory cascade.^(^^5^^)^ The neutrophil-lymphocyte ratio (NLR) is a prognostic marker used in different solid tumors,^(^^6^^–^^8^^)^ including GC,^(^^9^^)^ and the correlation between preoperative NLR and survival is widely reported.^(^^10^^)^ Systemic inflammatory status in the perioperative period is dynamic and influenced by the surgical procedure, physiological and psychological distress and tumoral mass removal.^(^^11^^)^ The proportion of inflammatory cells is modified after surgery, resulting in NLR variation. Thus, it is suggested that a more comprehensive evaluation could be done to estimate prognosis, rather than a single pre-operative NLR value. The NLR change analysis after curative surgery for GC may reflect this dynamic process, and its impact has not been established yet.

## OBJECTIVE

To evaluate the impact of neutrophil-lymphocyte ratio change on survival of patients with gastric cancer who underwent curative resection.

## METHODS

### Patients

**Figure 1:**
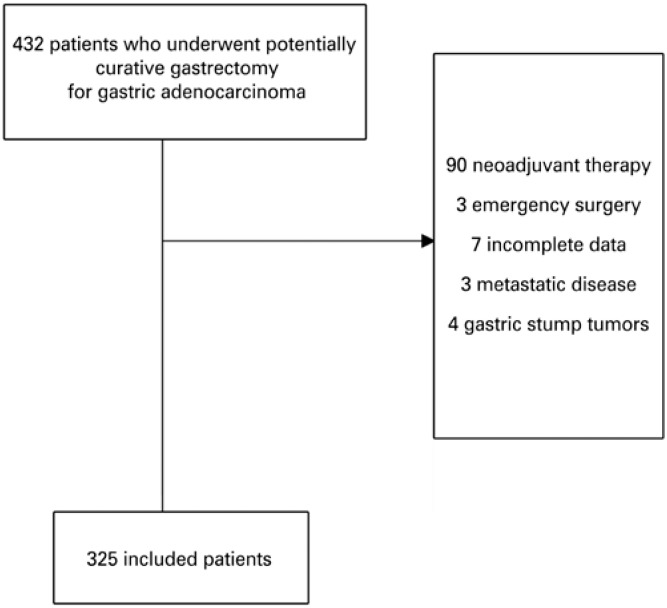
Flow chart of inclusion and exclusion criteria

A retrospective analysis of GC patients who underwent potentially curative gastrectomy between 2009 and 2017 was performed. All surgeries were performed by experienced teams who see a high volume of patients. Patients with histologically proven gastric adenocarcinoma and D1 or D2 lymphadenectomy were included. Patients with metastatic disease, gastric stump tumors, emergency surgery, neoadjuvant chemotherapy, or incomplete data in medical records were excluded (Figure 1). The surgical technique was performed following the guidelines of Japanese Gastric Cancer Association.^(^^12^^)^

Clinical characteristics studied included age, sex, body mass index (BMI), American Society of Anesthesiologists (ASA) preoperative risk score, and Charlson Comorbidity Index. Tumors were evaluated according to the TNM staging, eighth edition of the American Joint Committee on Cancer (AJCC) manual. Data were prospectively collected from a database. Postoperative complications were classified using the Clavien-Dindo classification.^(^^13^^)^ Postoperative mortality comprised patients who died within 30 days after surgical resection. Overall survival was defined as the interval from the surgery to death or last follow up, and disease-free survival (DFS) as the period from the surgery to first recurrence or final follow-up.

### Blood samples and neutrophil-lymphocyte ratio groups

Peripheral blood was obtained within 1 week before surgery, and at least 1 month after hospital discharge, to calculate pre and postoperative values, respectively.

Neutrophil-lymphocyte ratio was calculated by dividing the absolute count of neutrophils by that of lymphocytes, and its cutoff was established by the median value, separately for pre and postoperative periods. Low-high (LH) Groups represent NLR <cutoff and NLR ≥cutoff, respectively. Four subgroups were formed, as demonstrated in table 1.

**Table 1:** Subgroups according to neutrophil-lymphocyte ratio cutoff

Group	Preoperative	Postoperative	n (%)
	(cutoff =2.14)	(cutoff=1.8)	
LL	Low	Low	105 (32.3)
HL	High	Low	59 (18.1)
LH	Low	High	57 (17.5)
HH	High	High	104 (32)

High: patient neutrophil-lymphocyte ratio ≥ cut off value; low: patient neutrophil-lymphocyte ratio < cutoff value.

LL: low-low; HL: high-low; LH: low-high; HH: high-high.

### Statistical analysis

The clinicopathological characteristics were compared using χ^2^ tests for categorical variables, and analysis of variance (ANOVA - *análise de variância*) for continuous variables. Overall survival and DFS analyses were undertaken using the Kaplan-Meier method and the comparison of curves was obtained through the log-rank test. Multivariate analysis was performed by Cox proportional hazards to determine independent risk factors for overall survival. Only variables that were significant on univariate analysis were included as co-variables in the multivariable analyses. Survival time, in months, was calculated from the date of diagnosis until the date of death/recurrence. The patients alive were censored at the date of the last contact. The level of significance applied in the tests was of 5%, always considering the two-tailed alternative hypothesis. Software (SPSS), version 18.0 (SPSS Inc, Chicago, USA) was used for all analyses.

### Ethical considerations

The study was approved by the Internal Review Board of the *Faculdade de Medicina* of *Universidade de S*ão *Paulo*, under opinion number 2.286.610, CAAE: 76483517.8.0000.0065.

## RESULTS

### Baseline characteristics

A total of 325 patients met the inclusion criteria and were evaluated in this study. The mean age was 62.2 years (range from 25 to 88 years) and 198 (60.9%) were male. Subtotal gastrectomy and D2 lymphadenectomy were performed in most cases (64.3% and 88.6% of cases, respectively). The mean number of lymph nodes retrieved per surgical specimen was 39, and 186 (57.2%) patients had lymph node metastasis. The median pre and postoperative NLR was 2.14 (mean of 2.51; range from 0.41 to 16), and 1.80 (mean of 2.3; range from 0.13 to 28.8), respectively. Thus, the cutoff value used for the preoperative NLR was 2.14, and in the postoperative period, 1.80.

The subgroup division according to NLR change after surgery is shown in table 1, and the clinicopathological characteristics of the four groups are displayed in table 2.

**Table 2:** Clinical and pathological characteristics of gastric cancer patients according to the neutrophil-lymphocyte ratio subgroups

		LL NLR n=105 (32.3%)	HL NLR n=59 (18.1%)	LH NLR n=57 (17.5%)	HH NLR n=104 (32%)	p value
Age, years						0.008
	(SD)	58,9 (12,7)	64,6 (11,9)	62,3 (11,0)	64 (12,4)	
Sex						0.107
	Female	47 (44.8)	20 (33.9)	27 (47.4)	33 (31.7)	
	Male	58 (55.2)	39 (66.1)	30 (52.6)	71 (68.3)	
	BMI, kg/cm²	24.6 (4.4)	23.9 (5.5)	25.1 (5.5)	23.9 (4.5)	0.389
	Hemoglobin, g/dL	12.6 (2.0)	13.5 (16.4)	12.8 (1.8)	12 (2.2)	0.626
	Albumin, mg/dL	4.1 (0.7)	3.8 (0.7)	4.1 (0.5)	4.3 (3.1)	0.467
CCI*						0.454
	0	69 (65.7)	38 (64.4)	43 (75.4)	66 (63.5)	
	≥1	36 (34.3)	21 (35.6)	14 (24.6)	38 (36.5)	
ASA						0.054
	I/II	87 (82.9)	45 (76.3)	54 (94.7)	87 (83.7)	
	III/IV	18 (17.1)	14 (23.7)	3 (5.3)	17 (16.3)	
Tumor site						0.484
	Non-upper	98 (93.3)	54 (91.5)	49 (86)	94 (90.4)	
	Upper	7 (6.7)	5 (8.5)	8 (14)	10 (9.6)	
Type of resection						0.701
	Subtotal	28 (26.7)	16 (27.1)	11 (19.3)	28 (26.9)	
	Total	77 (73.3)	43 (72.9)	46 (80.7)	76 (73.1)	
Histological grade						0.676
	G1/G2	50 (47.6)	27 (45.8)	32 (56.1)	50 (48.1)	
	G3	55 (52.4)	32 (54.2)	25 (43.9)	54 (51.9)	
Lauren type						0.434
	Intestinal/ indeterminate	50 (47.6)	34 (57.6)	34 (59.6)	55 (52.9)	
	Diffuse/mixed	55 (52.4)	25 (42.4)	23 (40.4)	49 (47.1)	
pT status						0.086
	pT1/T2	50 (47.6)	19 (32.2)	28 (49.1)	37 (35.6)	
	pT3/T4	55 (52.4)	40 (67.8)	29 (50.9)	67 (64.4)	
pN status						0.569
	pN negative	44 (41.9)	21 (35.6)	27 (47.4)	47 (45.2)	
	pN positive	61 (58.1)	38 (64.4)	30 (52.6)	57 (54.8)	
Stage**						0.339
	I/II	55 (52.4)	29 (49.2)	37 (64.9)	57 (54.8)	
	III	50 (47.6)	30 (50.8)	20 (35.1)	47 (45.2)	
Postoperative complication					0.014
	No complication	81 (77.1)	39 (66.1)	34 (59.6)	80 (76.9)	
	Grade I-II	15 (14.3)	18 (30.5)	13 (22.8)	17 (16.3)	
	Grade III	9 (8.6)	2 (3.4)	10 (17.5)	7 (6.7)	

Results expressed as mean (standard deviation); n (%); mean (variation).

* Value without neoplasia and age;

** eighth edition of the American Joint Committee on Cancer (AJCC).

SD: standard deviation; BMI: body mass index; CCI: Charlson comorbidity index; ASA: American Society of Anesthesiologists; LL NLR: low pre and postoperative neutrophil-lymphocyte ratio; LH NLR: low pre and high postoperative neutrophil-lymphocyte ratio; HL NLR: high pre and low postoperative neutrophil-lymphocyte ratio; HH NLR: high pre and postoperative neutrophil-lymphocyte ratio.

Patients in Low-Low (LL) Control Group were younger as compared to the other NLR Groups (p=0.008). The frequency of ASA III/IV patients was higher in HL Group, however without reaching statistical significance. There was no difference in histological type, tumor invasion, lymph node metastases and tumor staging between groups.

In a mean follow-up of 31 months (median of 26.2; range 2.5-89.5 months), 94 patients presented disease recurrence and 92 died. The DFS and overall survival rates for the entire cohort were 71% and 70%, respectively. Mean length of hospital stay was of 11 days (range 4 to 59 days) and major complications (Clavien-Dindo >II) occurred in 28 (8.6%) cases. Severe postoperative complications were more common in the LH Group (p=0.014).

**Figure 2:**
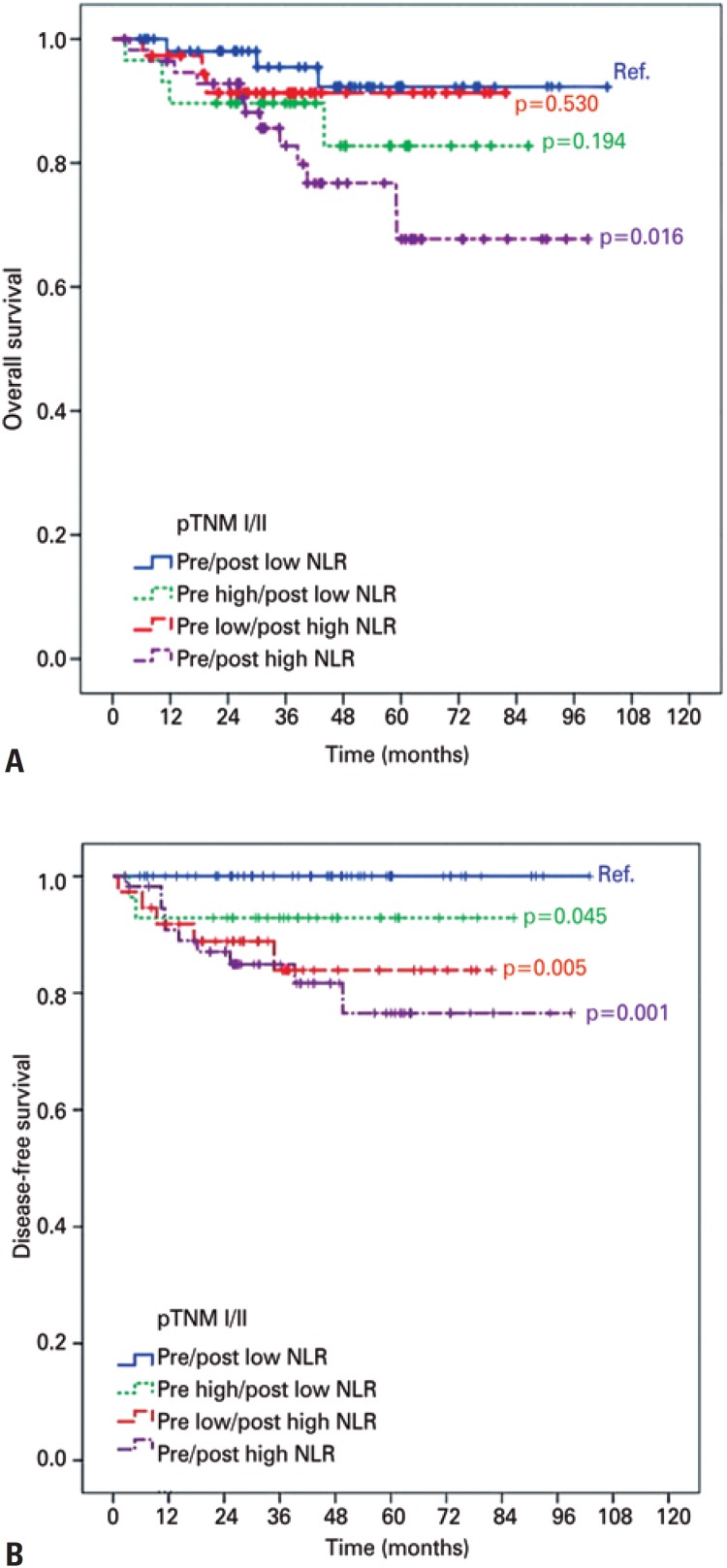
Kaplan-Meier curve comparing overall survival (A) and disease-free survival (B) according to neutrophil-lymphocyte ratio subgroup in stage I/II NLR: neutrophil-lymphocyte ratio.

In the survival analysis adjusted by staging, overall survival rate in pTNM I/II was worse for HH Group compared with LL Control Group (78.9% *versus* 94.5%; p=0.016) (Figure 2A). There was no difference in overall survival for stage III patients in HH Groups (p=0.115), LH Group (p=0.425) and HL Group (p=0.181), as compared to the Control Group LL. Concerning DFS, the HH, LH and HL Groups had worse rates compared with Control Group LL only in pTNM I/II (p=0.001, p=0.005 and p=0.045, respectively - Figure 2B). Also, there was no difference in DFS for HH Group (p=0.468), LH Group (p=0.425) and HL Group (p=0.375) groups compared to the Control Group LL pTNM III patients.

### Analysis of prognostic factors

Univariate and multivariate analyses were performed to evaluate the prognostic factors affecting overall survival in pTNM I/II patients. Charlson comorbidity index (CCI), pT status and HH NLR subgroup were identified as significant risk factors for poor survival in univariate analysis. In the multivariate analysis, CCI ≥1 and pT3/T4 were identified as independent prognostic factor for worse overall survival (Table 3).

**Table 3:** Overall survival analyses of pTNM I/II patients

Variables*		Univariate			Multivariate		
		Hazard ratio	95%CI	p value	Hazard ratio	95%CI	p value
Age 0-69 *versus* ≥70 years		2.19	0.95-5.06	0.066			
Female *versus* male		2.22	0.87-5.67	0.095			
Charlson 0-1 *versus* Charlson ≥1		2.32	1.01-5.38	0.049	2.52	1.05-6.08	0.039
Subtotal *versus* total gastrectomy		0.48	0.21-1.12	0.091			
D2 *versus* D1 gastrectomy		2.51	0.98-6.42	0.055			
pT1/2 *versus* pT3/4		2.85	1.23-6.57	0.014	3.09	1.29-7.42	0.011
pN0 *versus* pN+		1.84	0.75-4.53	0.182			
NLR subgroup LL (control)		1			1		
	HL	2.60	0.58-11.6	0.212	1.71	0.37-7.98	0.496
	LH	1.72	0.35-8.55	0.506	1.56	0.31-7.84	0.588
	HH	4.13	1.17-14.65	0.028	3.06	0.85-11.06	0.088

* Reference category listed first.

95%CI: confidence interval; NLR: neutrophil-lymphocyte ratio; LL: low-low; HL: high-low; LH: low-high; HH: high-high.

## DISCUSSION

Currently, the prognosis of patients with GC who underwent curative resection relies on pathologic and radiological analyses.^(^^14^^)^ It is noteworthy remembering that same stage disease patients may have different outcomes,^(^^15^^)^ raising the hypothesis that other factors may be involved in prognosis. In this context, serum inflammatory markers like NLR gain in importance, and could add clinical information that improves prognosis analysis.

Pre-operative NLR is established as a prognostic indicator in different solid tumors,^(^^9^^)^ however surgery and postoperative period lead to an inflammatory response resulting in change of blood cell proportion and NLR.^(^^11^^)^ Our rationale is that, since the perioperative period is dynamic regarding inflammatory changes, the evaluation of NLR in the postoperative context, added to the preoperative NLR, may reflect different prognostic scenarios.

Thus, we performed this study to clarify the precise value of NLR before and after surgery. Our results suggest that a high NLR in pre and/or postoperative period (HH, LH and HL Groups) are significantly correlated with lower survival in stage I/II GC, compared with LL Control Group.

Most studies regarding NLR focus on a single pre-operative value,^(^^10^^,^^16^^)^ which seems to reflect the balance between inflammatory and antitumor status. Our group has already described in a previous study that high preoperative NLR levels correlate with worse prognosis in GC patients.^(^^17^^)^ However, data regarding NLR change after tumor resection is scarce.

The few studies available generally demonstrate a worse prognosis when NLR increases after surgery in different types of solid tumors,^(^^18^^–^^20^^)^ including GC.^(^^21^^)^ The present research classified patients according to their median NLR values in low and high NLR Groups, for both pre and postoperative periods, which provided four subgroups Dan et al., studying patients with hepatocarcinoma treated with radioablation, demonstrated that subgroup change after treatment correlates with different overall survival and DFS.^(^^22^^)^

Based on the median value, the cutoff established for NLR before and after gastrectomy was 2.14 and 1.8, respectively. This decrease may suggest that there is a tendency in the reduction of the systemic inflammatory response after surgery, pointing out that tumor mass itself enhances inflammation. The current research also investigated the relation between NLR Subgroups and clinical and pathological characteristics. No difference was observed in relation to prognostic parameters, such as tumor invasion and lymph node metastases.

However, we observed a higher frequency of younger patients in Control Group LL (p=0.008) and the occurrence of severe postoperative complications in LH Subgroup (p=0.014). Our hypothesis is that inflammatory response to severe complications persists even one month after hospital discharge and is reflected in the increase of NLR value. This finding was first demonstrated by Cook et al., in colorectal surgery, who related high NLR in the immediate postoperative period, with increased risk of complications.^(^^23^^)^ Regarding age, it can be justified by the fact that younger patients usually have better antitumor response than older patients with depressed immune system.^(^^24^^,^^25^^)^

Although not significant, we observed a higher frequency of more advanced cases in the HL Group, as well as the predominance of less advanced patients in the LH Group, which corresponds to the group associated with the occurrence of complications. This may raise the hypothesis that preoperative NLR reflects more tumor status (higher in more advanced states), whereas postoperative NLR seems to reflect more patient status (such as the occurrence of surgical complications), and not only the presence of tumor cells remaining after surgical resection.^(^^26^^)^

A clinically relevant finding of this research was that NLR Subgroups correlated with overall survival and DFS in less advanced stages (pTNM I/II). The HH Subgroup presented the worse overall survival (p=0.016) and DFS (p = 0.001) compared to the LL Subgroup; and LH and HL Groups had lower SLD (p=0.005 and p=0.045, respectively), but not overall survival. Although there were no significant differences in survival for pTNM III GC, this finding allows risk stratification among NLR Subgroups. A possible explanation is the fact that inflammation is more exacerbated in patients with stage III disease, making risk stratification by inflammatory markers more difficult in advanced stages. In addition, the presence of residual tumor cells secreting pro-tumoral cytokines may justify the worse prognosis when a high NLR persists. This hypothesis was also highlighted by Miyatani et al.,^(^^27^^)^ and may partially elucidate why postoperative NLR value is relevant.

Although worse survival rates were observed in HH NLR Group, high levels of NLR were not an independent factor associated with survival in multivariate analysis. This result diverges from the available data,^(^^21^^)^ where positive NLR changes were associated with worse DFS (hazard ratio − HR=2.53; 95%CI: 1.8-3.56; p<0.001) and overall survival (HR=1.45; 95%CI: 1.06-1.97; p<0.001). This difference may be attributed to the greater number of patients included in the study (n=734), in addition to the fact that most cases were stage I/II (72.6%).

Some limitations should be considered in the present study. This is a retrospective study, conducted in a single center. There is a lack of consensus regarding the cut-off value of the NLR and best postoperative period to obtain blood samples.^(^^16^^)^ Some studies used Receiver Operating Characteristics (ROC) curve instead of median values. Moreover, some assessed the difference between the pre and post NLR values, while others considered the NLR periods separately, restricting the generalizability of our results. Inflammation due to surgical procedure certainly alters NLR values^(^^11^^)^ and might mask its desired function of reflecting tumoral microenvironment. Thus, it is reasonable to avoid an early postoperative assessment of NLR value. Another limitation is the small sample size, which also limits the generalization of the results.

Overall, this study showed that NLR change after surgery may help to stratify risk in stages I and II. These results strengthen the idea that NLR is an easy and cost-effective method to predict overall survival and DFS, and provide more individualized information about prognosis and survival of GC patients. Moreover, LH subgroup was associated with the occurrence of postoperative complications. Hence, strategies to enhance prognosis evaluation may involve the utilization of perioperative NLR rather than a single pre-operative NLR value.

In addition, other laboratory parameters, such as C-reactive protein, should be evaluated in association with the NLR to better characterize patient status, helping to identify potential prognostic markers that can together provide more accurate information about their real impact on survival.

## CONCLUSION

Pre and postoperative neutrophil-lymphocyte ratio value may assist risk stratification in patients with gastric cancer stage I or II. The high-high, high-low and low-high neutrophil-lymphocyte ratio values were significantly associated with worse disease-free survival, compared with low-low values; and lower overall survival rate was associated with high-high neutrophil-lymphocyte ratio. Also, the low-high subgroup was related with the occurrence of postoperative complications. Neutrophil-lymphocyte ratio is a useful marker and should be adopted routinely in prognostic evaluation.
