# Supplementary material for: Neutrophil-lymphocyte ratio change after curative gastrectomy for gastric cancer: a subgroup analysis
Source: Einstein (Sao Paulo). 2019 Nov 14;18:eAO4860. doi: 10.31744/einstein_journal/2020AO4860 (PMC6896601; doi:10.31744/einstein_journal/2020AO4860)
Supplement: Translation

# Translation: Evolução da relação neutrófilo-linfócito após gastrectomia curativa por câncer gástrico: análise de subgrupos

**Authors:** Daniel José Szor, André Roncon Dias, Marina Alessandra Pereira, Marcus Fernando Kodama Pertille Ramos, Bruno Zilberstein, Ivan Cecconello, Ulysses Ribeiro

**Affiliations:** 1 São PauloSP Brasil Instituto do Câncer do Estado de São Paulo, Faculdade de Medicina, Universidade de São Paulo, São Paulo, SP, Brasil.

**Keywords:** Neoplasias gástricas, Prognóstico, Biomarcadores, Inflamação, Gastrectomia

## Abstract

**Objetivo:**

Avaliar o impacto da alteração da relação neutrófilo-linfócito após ressecção curativa por câncer gástrico.

**Métodos:**

Realizou-se análise retrospectiva de pacientes com câncer gástrico submetidos à gastrectomia curativa entre 2009 e 2017. Foi estabelecido valor de corte para a relação neutrófilo-linfócito nos períodos pré e pós-operatório de acordo com a mediana, e quatro subgrupos foram formados (baixo-baixo/baixo-alto/alto-baixo/alto-alto). Dados clínicos e patológicos e de sobrevida foram analisados e relacionados com estes subgrupos.

**Resultados:**

Foram incluídos no estudo 325 pacientes. Os valores de corte para a relação neutrófilo-linfócito foram 2,14 e 1,8 para os períodos pré e pós-operatório, respectivamente. Em pacientes com estádios I e II, o subgrupo alto-alto apresentou pior sobrevida global (p=0,016) e sobrevida livre de doença (p=0,001). As complicações ocorreram mais em pacientes do subgrupo baixo-alto.

**Conclusão:**

A relação neutrófilo-linfócito é um marcador de baixo custo, eficiente e reprodutível. A individualização do prognóstico pode ser realizada de acordo com a identificação de subgrupos com maior risco de complicações e pior prognóstico.

## INTRODUÇÃO

O câncer gástrico (CG) é a quinta neoplasia mais comum e a terceira maior causa de mortes por câncer no mundo.^(^^1^^)^ Pacientes com o mesmo estágio da doença podem ter desfechos diferentes, o que sugere que vários fatores podem contribuir para o prognóstico. A relação entre inflamação e câncer já é conhecida desde o século 19^(^^2^^)^ e, na última década, propôs-se o uso de biomarcadores inflamatórios para refinar o prognóstico.^(^^3^^,^^4^^)^ O microambiente tumoral é formado por múltiplos tipos celulares, incluindo neutrófilos e linfócitos, que têm funções específicas na cascata inflamatória.^(^^5^^)^ A relação neutrófilo-linfócito (RNL) é um marcador prognóstico utilizado em diferentes tumores sólidos,^(^^6^^–^^8^^)^ inclusive o CG,^(^^9^^)^ e a correlação entre a RNL no pré-operatório e a sobrevida já foi amplamente reportada.^(^^10^^)^ O estado inflamatório sistêmico no período perioperatório é dinâmico e influenciado por procedimento cirúrgico, perturbações fisiológicas e psicológicas, e pela remoção da massa tumoral.^(^^11^^)^ A proporção de células inflamatórias muda após a cirurgia, o que resulta em uma variação da RNL. Desse modo, é possível que uma avaliação mais abrangente possa ser feita, para estimar o prognóstico, em vez de simplesmente se utilizar o valor pré-operatório da RNL. A análise da evolução da RNL após cirurgia curativa para CG pode refletir esse processo dinâmico, e seu impacto ainda não foi estabelecido.

## OBJETIVO

Avaliar o impacto da evolução da relação neutrófilo-linfócito na sobrevida de pacientes com câncer gástrico submetidos à ressecção curativa.

## MÉTODOS

### Pacientes

Foi realizada uma análise de pacientes com CG submetidos à gastrectomia potencialmente curativa, entre 2009 e 2017. Todas as cirurgias foram realizadas por equipes experientes, que atendem um alto volume. Foram incluídos pacientes com adenocarcinoma gástrico comprovado histologicamente e linfadenectomia D1 ou D2. Foram excluídos pacientes com doença metastática, tumores do coto gástrico, cirurgia de emergência, quimioterapia neoadjuvante ou dados incompletos nos prontuários médicos (Figura 1). A técnica cirúrgica foi realizada de acordo com as diretrizes da *Japanese Gastric Cancer Association*.^(^^12^^)^

**Figura 1:** Fluxograma de critérios de inclusão e exclusão

As características clínicas estudadas incluíram idade, sexo, índice de massa corporal (IMC), escore de risco pré-operatório da *American Society of Anesthesiologists* (ASA) e índice de comorbidades de Charlson. Os tumores foram avaliados de acordo com o estadiamento TNM que consta na oitava edição do manual do *American Joint Committee on Cancer* (AJCC). Os dados foram coletados prospectivamente a partir de um banco de dados. As complicações pós-operatórias foram classificadas pela escala de Clavien-Dindo.^(^^13^^)^ A mortalidade pós-operatória incluiu pacientes que vieram a óbito até 30 dias após a ressecção cirúrgica. A sobrevida global (SG) foi definida como o intervalo da cirurgia até o óbito ou último acompanhamento, e a sobrevida livre de doença (SLD) como o intervalo da cirurgia até a primeira recidiva ou o acompanhamento final.

### Amostras de sangue e grupos de relação neutrófilo-linfócito

Sangue periférico foi obtido na semana anterior à cirurgia e no mínimo 1 mês após a alto hospitalar, para calcular os valores pré e pós-operatórios, respectivamente.

A RNL foi calculada pela divisão da contagem absoluta de neutrófilos pela de linfócitos, e o valor de corte foi o valor mediano para os períodos pré e pós-operatório, separadamente. Os Grupos Baixo-Alto (BA) representaram RNL < valor de corte e RNL ≥ valor de corte, respectivamente. Quatro subgrupos foram formados, conforme demonstrado na tabela 1.

**Tabela 1:** Subgrupos de acordo com ponto de corte da relação neutrófilo-linfócito

Grupo	Pré-operatório	Pós-operatório	n (%)
	(Ponto de corte = 2,14)	(Ponto de corte = 1,8)	
**BB**	Baixo	Baixo	105 (32,3)
**AB**	Alto	Baixo	59 (18,1)
**BA**	Baixo	Alto	57 (17,5)
**AA**	Alto	Alto	104 (32)

Alto: razão neutrófilo-linfócito do paciente ≥ valor de corte; baixo: razão neutrófilo-linfócito do paciente < valor de corte.

BB: baixo-baixo; AB: alto-baixo; BA: baixo-alto; AA: alto-alto.

### Análise estatística

As características clínico-patológicas foram comparadas por meio dos testes χ^2^ para variáveis categóricas, e análise de variância (ANOVA), para variáveis contínuas. As análises de SG e SLD foram realizadas pelo método de Kaplan-Meier e a comparação das curvas foi feita pelo teste de *log-rank*. Uma análise multivariada de riscos proporcionais de Cox foi realizada para determinar os fatores de risco independentes para a SG. Apenas as variáveis que foram significativas na análise univariada foram incluídas como covariáveis nas análises multivariadas. O tempo de sobrevida, em meses, foi calculado a partir da data do diagnóstico até a data do óbito/recidiva. Os pacientes vivos foram recenseados na data do último contato. O nível de significância aplicado nos testes foi de 5%, sempre considerando a hipótese alternativa bicaudal. O programa (SPSS), versão 18.0. (SPSS Inc, Chicago, EUA) foi usado em todas as análises.

### Considerações éticas

O estudo foi aprovado pelo Comitê de Ética da Faculdade de Medicina da Universidade de São Paulo, sob o parecer 2.286.610, CAAE: 76483517.8.0000.0065.

## RESULTADOS

### Características iniciais

Um total de 325 pacientes preencheu os critérios de inclusão e foi avaliado neste estudo. A média de idade foi 62,2 anos (variação de 25 a 88 anos), e 198 (60,9%) eram homens. Foram realizadas gastrectomia subtotal e linfadenectomia D2 na maioria dos casos (64,3% e 88,6% dos casos, respectivamente). O número médio de linfonodos removidos por cada peça cirúrgica foi de 39, e 186 (57,2%) pacientes tinham metástases linfonodais. O valor mediano da RNL no pré e pós-operatório foi de 2,14 (média de 2,51; variação de 0,41 a 16) e 1,80 (média de 2,3; variação de 0,13 a 28,8), respectivamente. O valor de corte utilizado para a RNL no pré-operatório foi 2,14 e, no pós-operatório, 1,80.

A divisão em subgrupos de acordo com a evolução da RNL após a cirurgia é mostrada na tabela 1, e as características clínico-patológicas dos quatro grupos são apresentadas na tabela 2.

**Tabela 2:** Características clínico-patológicas dos pacientes com câncer gástrico, de acordo com subgrupos da relação neutrófilo-linfócito

Variáveis		RNL BB n=105 (32,3%)	RNL AB n=59 (18,1%)	RNL BA n=57 (17,5%)	RNL AA n=104 (32%)	Valor de p
Idade (anos)						0,008
	(DP)	58,9 (12,7)	64,6 (11,9)	62,3 (11,0)	64 (12,4)	
Sexo						0,107
	Feminino	47 (44,8)	20 (33,9)	27 (47,4)	33 (31,7)	
	Masculino	58 (55,2)	39 (66,1)	30 (52,6)	71 (68,3)	
IMC (Kg/cm²)						0,389
	Média (variação)	24,6 (4,4)	23,9 (5,5)	25,1 (5,5)	23,9 (4,5)	
Hemoglobina (g/dL)						0,626
	Média (DP)	12,6 (2,0)	13,5 (16,4)	12,8 (1,8)	12 (2,2)	
Albumina (mg/dL)						0,467
	Média (DP)	4,1 (0,7)	3,8 (0,7)	4,1 (0,5)	4,3 (3,1)	
ICC*						0,454
	0	69 (65,7)	38 (64,4)	43 (75,4)	66 (63,5)	
	>1	36 (34,3)	21 (35,6)	14 (24,6)	38 (36,5)	
ASA						0,054
	I/II	87 (82,9)	45 (76,3)	54 (94,7)	87 (83,7)	
	III/IV	18 (17,1)	14 (23,7)	3 (5,3)	17 (16,3)	
Local do tumor						0,484
	Não trato superior	98 (93,3)	54 (91,5)	49 (86)	94 (90,4)	
	Trato superior	7 (6,7)	5 (8,5)	8 (14)	10 (9,6)	
Tipo de ressecção						0,701
	Subtotal	28 (26,7)	16 (27,1)	11 (19,3)	28 (26,9)	
	Total	77 (73,3)	43 (72,9)	46 (80,7)	76 (73,1)	
Classificação histológica						0,676
	G1/G2	50 (47,6)	27 (45,8)	32 (56,1)	50 (48,1)	
	G3	55 (52,4)	32 (54,2)	25 (43,9)	54 (51,9)	
Classificação de Lauren						0,434
	Intestinal/indeterminado	50 (47,6)	34 (57,6)	34 (59,6)	55 (52,9)	
	Difuso/misto	55 (52,4)	25 (42,4)	23 (40,4)	49 (47,1)	
Estado pT						0,086
	pT1/T2	50 (47,6)	19 (32,2)	28 (49,1)	37 (35,6)	
	pT3/T4	55 (52,4)	40 (67,8)	29 (50,9)	67 (64,4)	
Estado pN						0,569
	pN negativo	44 (41,9)	21 (35,6)	27 (47,4)	47 (45,2)	
	pN positivo	61 (58,1)	38 (64,4)	30 (52,6)	57 (54,8)	
Estágio**						0,339
	I/II	55 (52,4)	29 (49,2)	37 (64,9)	57 (54,8)	
	III	50 (47,6)	30 (50,8)	20 (35,1)	47 (45,2)	
Complicação pós-operatória						0,014
	Sem complicação	81 (77,1)	39 (66,1)	34 (59,6)	80 (76,9)	
	Grau I-II	15 (14,3)	18 (30,5)	13 (22,8)	17 (16,3)	
	Grau III	9 (8,6)	2 (3,4)	10 (17,5)	7 (6,7)	

Resultados expressos por média (desvio padrão); n (%): média (variação).

* Valor sem neoplasia e idade;

** oitava edição do Manual da *American Joint Committee on Cancer*.

DP: desvio padrão; IMC: índice de massa corporal; ICC: índice de comorbidade de Charlson; ASA: *American Society of Anesthesiologists*; RNL BB: relação neutrófilo-linfócito baixa no pré e baixa pós-operatório; RNL BA: relação neutrófilo-linfócito baixa no pré e alta no pós-operatório; RNL AB: relação neutrófilo-linfócito alta no pré e baixa no pós-operatório; RNL AA: relação neutrófilo-linfócito alta no pré e alta no pós-operatório.

Os pacientes do Grupo Controle Baixo-Baixo (BB) eram mais jovens, em comparação aos outros Grupos de RNL (p=0,008). A frequência de pacientes ASA III/IV foi mais alta no Grupo AB, entretanto sem significância estatística. Não houve diferença no tipo histológico, invasão tumoral, metástases linfonodais e estadiamento dos tumores entre os grupos.

Em um acompanhamento médio de 31 meses (mediana de 26,2; variação de 2,5 a 89,5 meses), 94 pacientes tiveram recidiva da doença, e 92 vieram a óbito. As taxas de SLD e SG de toda a coorte foram 71% e 70%, respectivamente. O tempo médio de internação foi de 11 dias (variando de 4 a 59 dias), e complicações maiores (Clavien-Dindo >II) ocorreram em 28 (8,6%) casos. Complicações graves no pós-operatório foram mais comuns no Grupo BA (p=0,014).

Na análise de sobrevida ajustada por estadiamento, a taxa de SG para pTNM I/II foi pior no Grupo AA em comparação ao Grupo Controle BB (78,9% *versus* 94,5%; p=0,016) (Figura 2A). Não houve diferença na SG para pacientes em estágio III dos Grupos AA (p=0,115), Grupo BA (p=0,425) e Grupo AB (p=0,181), em comparação ao Grupo Controle BB. Em relação à SLD, os Grupos AA, BA e AB tiveram taxas piores em comparação ao Grupo Controle BB apenas para pTNM I/II (p=0,001; p=0,005 e p=0,045, respectivamente; Figura 2B). Além disso, não houve diferença na SLD para os Grupos AA (p=0,468), Grupo BA (p=0,425) e Grupo AB (p=0,375), em comparação ao Grupo Controle BB, para pacientes pTNM III.

**Figura 2:** Curva de Kaplan-Meier comparando sobrevida global (A) e sobrevida livre de doença (B), de acordo com o subgrupo da relação linfócito-neutrófilo para estágios I/II RLN: relação linfócito-neutrófilo.

### Análise de fatores prognósticos

Foram realizadas análises univariada e multivariada para avaliar os fatores prognósticos que afetam a SG em pacientes pTNM I/II. O Índice de Comorbidade de Charlson (ICC), o *status* pT e o Subgrupo RNL AA foram identificados como fatores de risco significativos para pior sobrevida na análise univariada. Na análise multivariada, ICC ≥1 e pT3/T4 foram identificados como fatores prognósticos independentes para uma pior SG (Tabela 3).

**Tabela 3:** Análises de sobrevida global de pacientes com pTNM I/II

Variáveis*		Univariada			Multivariada		
		*Hazard ratio*	IC95%	Valor de p	*Hazard ratio*	IC95%	Valor de p
Idade 0-69 *versus* ≥70 anos		2,19	0,95-5,06	0,066			
Feminino *versus* masculino		2,22	0,87-5,67	0,095			
Charlson 0-1 *versus* Charlson ≥1		2,32	1,01-5,38	0,049	2,52	1,05-6,08	0,039
Gastrectomia subtotal *versus* total		0,48	0,21-1,12	0,091			
Gastrectomia D2 *versus* D1		2,51	0,98-6,42	0,055			
pT1/2 *versus* pT3/4		2,85	1,23-6,57	0,014	3,09	1,29-7,42	0,011
pN0 *versus* pN+		1,84	0,75-4,53	0,182			
Subgrupo RNL BB (controle)		1			1		
	AB	2,60	0,58-11,6	0,212	1,71	0,37-7,98	0,496
	BA	1,72	0,35-8,55	0,506	1,56	0,31-7,84	0,588
	AA	4,13	1,17-14,65	0,028	3,06	0,85-11,06	0,088

* Categoria de referência listada em primeiro lugar.

IC95%: intervalo de confiança; RNL: relação neutrófilo-linfócito; BB: baixo-baixo; AB: alto-baixo; BA: baixo-alto; AA: alto-alto.

## DISCUSSÃO

Atualmente, o prognóstico de pacientes com CG submetidos à ressecção curativa depende de análise patológica e radiológica.^(^^14^^)^ É importante lembrar que pacientes com o mesmo estágio da doença podem ter desfechos diferentes,^(^^15^^)^ o que levanta a hipótese de que outros fatores podem estar envolvidos no prognóstico. Nesse contexto, marcadores inflamatórios séricos, como a RNL, ganham importância e poderiam trazer informações adicionais para melhorar a análise de prognóstico.

A RNL pré-operatória é um indicador prognóstico estabelecido em alguns tumores sólidos,^(^^9^^)^ mas a cirurgia e o pós-operatório levam a uma resposta inflamatória que resulta em alterações na proporção de células do sangue e, consequentemente, na RNL.^(^^11^^)^ Nosso raciocínio é que, como o período perioperatório é dinâmico em relação a alterações inflamatórias, a avaliação da RNL no contexto pós-operatório, além da RNL pré-operatória, pode refletir diferentes cenários prognósticos.

Assim, conduzimos este estudo para esclarecer qual é precisamente a relevância da RNL antes e depois da cirurgia. Nossos resultados sugerem que RNL alta no pré e/ou pós-operatório (Grupos AA, BA e AB) tem correlação considerável com sobrevida mais baixa para CG estágio I/II, em comparação ao Grupo Controle BB.

A maioria dos estudos sobre RNL foca-se em um único valor pré-operatório,^(^^10^^,^^16^^)^ que parece refletir o equilíbrio entre os estados inflamatório e antitumoral. Nosso grupo já descreveu, em estudo anterior, que valores altos de RNL pré-operatória têm correlação com piores prognósticos em pacientes com CG.^(^^17^^)^ No entanto, ainda há poucos dados relativos à evolução da RNL após a ressecção do tumor.

Os poucos estudos disponíveis geralmente demonstram piores prognósticos quando a RNL aumenta após a cirurgia em diferentes tipos de tumores sólidos,^(^^18^^–^^20^^)^ incluindo CG.^(^^21^^)^ O estudo atual classificou os pacientes de acordo com as medianas de RNL nos Grupos RNL alta e baixa, no pré e pós-operatório, resultando em quatro subgrupos. Dan et al., ao estudarem pacientes com hepatocarcinoma tratados com radioablação, demonstraram que a mudança de subgrupo após o tratamento tem correlação com diferenças na SG e SLD.^(^^22^^)^

Com base na mediana, o valor de corte estabelecido para a RNL antes e depois da gastrectomia foi de 2,14 e 1,8, respectivamente. Essa redução pode indicar que existe uma tendência de diminuição da resposta inflamatória sistêmica após a cirurgia, sugerindo que a massa tumoral propriamente dita talvez aumente a inflamação. Nosso estudo também investigou a relação entre os Subgrupos de RNL e as características clínico-patológicas. Não foi observada diferença em relação aos parâmetros prognósticos, como invasão tumoral e metástases linfonodais.

No entanto, observamos frequência mais alta de pacientes mais jovens no Grupo Controle BB (p=0,008), bem como a ocorrência de complicações pós-operatórias graves no Subgrupo BA (p=0,014). Nossa hipótese é a de que a resposta inflamatória a complicações graves persiste até 1 mês após a alta hospitalar, e isso se reflete no valor da RNL. Esse achado foi primeiramente demonstrado por Cook et al., na cirurgia colorretal, tendo sido encontrada relação entre alto valor de RNL no pós-operatório imediato e maior risco de complicações.^(^^23^^)^ Em relação à idade, a justificativa pode estar no fato de que pacientes mais jovens geralmente têm melhor resposta antitumoral do que pacientes mais velhos com o sistema imunológico deprimido.^(^^24^^,^^25^^)^

Embora sem significância, observamos frequência mais alta de casos mais avançados no Grupo AB, bem como predominância de pacientes menos avançados no Grupo BA, que corresponde ao grupo associado com a ocorrência de complicações. Isso pode levantar a hipótese de que a RNL pré-operatória refletiria mais o estado do tumor (mais alto em estados mais avançados), enquanto a RNL pós-operatória refletiria mais o estado do paciente (como, por exemplo, a ocorrência de complicações cirúrgicas), e não apenas a presença de células tumorais remanescentes após a ressecção cirúrgica.^(^^26^^)^

Um achado clinicamente relevante deste estudo foi que os Subgrupos de RNL tiveram correlação com a SG e a SLD em estágios menos avançados (pTNM I/II). O Subgrupo AA apresentou piores SG (p=0,016) e SLD (p=0,001) em comparação ao Subgrupo BB; e os Grupos BA e AB apresentaram SLD mais baixo (p=0,005 e p=0,045, respectivamente), mas não SG. Embora não tenha havido diferenças significativas na sobrevida para CG pTNM III, esse achado permite a estratificação do risco entre os Subgrupo de RNL. Uma explicação possível é o fato de a inflamação ser mais exacerbada em pacientes com estágio III da doença, o que torna a estratificação de risco por marcadores inflamatórios mais difícil em estágios mais avançados. Além disso, a presença de células tumorais residuais secretando citocinas pró-tumorais pode justificar o pior prognóstico quando há persistência de valores altos de RNL. Essa hipótese também foi destacada por Miyatani et al.,^(^^27^^)^ e pode explicar parcialmente por que o valor pós-operatório da RNL é relevante.

Apesar das piores taxas de sobrevida observadas no Grupo de RNL AA, altos valores de RNL não foram fator independente associado à sobrevida na análise multivariada. Esse resultado diverge dos dados disponíveis,^(^^21^^)^ em que variações positivas na RNL foram associadas a valores piores de SLD (*hazard ratio* − HR=2,53; IC95%: 1,8-3,56; p<0,001) e SG (HR=1,45; IC95%: 1,06-1,97; p<0,001). Esta diferença pode ser atribuída ao maior número de pacientes incluídos no estudo (n=734), além do fato de a maioria dos casos ser estágios I/II (72,6%).

Algumas limitações devem ser consideradas no presente estudo. Este é um estudo retrospectivo, conduzido em um único centro. Há uma falta de consenso sobre o valor de corte da RNL e o melhor momento no pós-operatório para a obtenção de amostras de sangue.^(^^16^^)^ Alguns estudos usaram a curva Características de Operação do Receptor (COR) em vez dos valores da mediana. Além disso, alguns avaliaram a diferença entre os valores pré e pós-operatórios de RNL, enquanto outros consideraram os períodos de RNL separadamente, restringindo a possibilidade de generalização de nossos resultados. A inflamação decorrente do procedimento cirúrgico certamente altera os valores de RNL^(^^11^^)^ e pode mascarar a função desejável para esses valores, que é de refletir o microambiente tumoral. Assim, é recomendável que se evite fazer avaliação precoce do valor da RNL no pós-operatório. Outra limitação é o pequeno tamanho da amostra, o que também limita a generalização dos resultados.

No geral, este estudo mostrou que a evolução da RNL após a cirurgia pode ajudar a estratificar o risco nos estágios I e II. Esses resultados reforçam a ideia de que a RNL é um método fácil e custo-efetivo de prever a SG e a SLD, e fornecer informações mais individualizadas sobre o prognóstico e a sobrevida dos pacientes com CG. Além disso, o Subgrupo BA foi associado à ocorrência de complicações pós-operatórias. Portanto, possíveis estratégias para melhorar a avaliação prognóstica podem envolver a utilização da RNL perioperatória, em vez de apenas um valor de RNL pré-operatória.

Ademais, outros parâmetros laboratoriais, como a proteína C-reativa, devem ser avaliados em associação à RNL, para melhor caracterização do estado do paciente, ajudando a identificar potenciais marcadores prognósticos que, juntos, possam fornecer informações mais precisas sobre seu verdadeiro impacto na sobrevida.

## CONCLUSÃO

A relação neutrófilo-linfócito no pré e pós-operatório pode ajudar na estratificação de risco em pacientes com câncer gástrico em estágio I ou II. Os valores de relação neutrófilo-linfócito alto-alto, alto-baixo e baixo-alto foram consideravelmente associados a uma pior sobrevida livre de doença, em comparação aos valores baixo-baixo; e uma sobrevida global mais baixa foi associada a valores de relação neutrófilo-linfócito alto-alto. Além disso, o subgrupo baixo-alto foi relacionado com a ocorrência de complicações pós-operatórias. A relação neutrófilo-linfócito é um marcador útil e deve ser adotada rotineiramente para avaliação de prognóstico.
